# Metal Dichalcogenides Monolayers: Novel Catalysts for Electrochemical Hydrogen Production

**DOI:** 10.1038/srep05348

**Published:** 2014-06-26

**Authors:** Hui Pan

**Affiliations:** 1 Institute of Applied Physics and Materials Engineering, Faculty of Science and Technology, University of Macau, Macau SAR, China

## Abstract

Catalyst-driven electrolysis of water is considered as a “cleanest” way for hydrogen production. Finding cheap and abundant catalysts is critical to the large-scale implementation of the technology. Two-dimensional metal dichalcogenides nanostructures have attracted increasing attention because of their catalytic performances in water electrolysis. In this work, we systematically investigate the hydrogen evolution reduction of metal dichalcogenides monolayers based on density-functional-theory calculations. We find that metal disulfide monolayers show better catalytic performance on hydrogen production than other metal dichalcogenides. We show that their hydrogen evolution reduction strongly depends on the hydrogen coverage and the catalytic performance reduces with the increment of coverage because of hydrogenation-induced lower conductivity. We further show that the catalytic performance of vanadium disulfide monolayer is comparable to that of Pt at lower hydrogen coverage and the performance at higher coverage can be improved by hybridizing with conducting nanomaterials to enhance conductivity. These metal disulfide monolayers with lower overpotentials may apply to water electrolysis for hydrogen production.

Energy has been a key in the development of every sphere of human society. The traditional usable energy sources, including fossil fuels and coal, will fall short of the demand of sustainable development over the long term, and their continued use produces harmful side effects such as pollution that threatens human health and greenhouse gases associated with climate change, which have triggered considerable world-wide effort to explore renewable green energy alternatives^1^. Hydrogen is considered to be one of the most important candidates because of its abundance and clean and renewable nature. As an ideal clean energy carrier for future, hydrogen can be produced from a variety of energy resources, has the highest energy density per unit mass, and produce the least polluting since it can be extracted from natural resource such as water or biomass and its use produces water only. The electrolysis of water is considered as a well-known principle to produce oxygen and hydrogen gas in a sustainable fashion^2^,^3^,^4^,^5^,^6^. The key component in electrochemical reduction of water is the catalyst for hydrogen evolution reduction (HER). The well-known catalysts in the electrolysis of water are noble metals, such as platinum, due to their superior electrocatalytic properties^7^,^8^,^9^,^10^. The application of noble metal catalysts in large-scale production of hydrogen is limited by their high cost and low abundance, although lots of efforts had been done by tuning the composition of the catalyst to modify their electronic structures^11^,^12^,^13^,^14^,^15^,^16^,^17^,^18^,^19^,^20^,^21^,^22^,^23^,^24^. On the other hands, considerable efforts have been carried out to search alternative catalysts with lower cost and abundance^22^,^23^,^24^,^25^. Basically, an advanced catalyst for the enhanced electrochemical hydrogen evolution reaction should reduce the HER reaction overpotential and consequently increase the HER efficiency^3^,^4^,^5^,^6^,^25^.

The transition-metal dichalcogenides with the formula of MX_2_, where M is a transition metal element from group IV (Ti, Zr, or Hf), group V (for instance V, Nb or Ta) or group VI (Mo or W), and X is a chalcogen (S, Se or Te), have attracted increasing attention for their applications in electrolysis of water^26^,^27^,^28^,^29^,^30^,^31^,^32^,^33^,^34^,^35^,^36^,^37^,^38^,^39^,^40^,^41^,^42^,^43^. These materials have crystal structures consisting of weakly coupled sandwich layers *X*-M-*X*, where one M-atom layer is enclosed within two X layers and the atoms in layers are hexagonally packed^43^. The overall symmetry of transition metal dichalcogenides can be hexagonal or rhombohedral, and the metal atoms have octahedral (1T) or trigonal prismatic (2H) coordination^43^. Experimental and theoretical studies had suggested that the electrocatalytical activity of 2H-MoS_2_ in electrolysis of water is contributed to its edges^27^,^28^,^29^, which are metallic if they are zigzag^44^,^45^. Recently, Voiry et al.^37^ reported that metallic 1T-WS_2_ nanosheets showed better HER performance than semiconducting 2H-WS_2_, which can be further improved by strain engineering. As compared with the noble metals, we may be sure that only MX_2_ monolayers with high conductivity, and MX_2_ nanoribbons/nanoparticles with metallic edges can promise the excellent HER activity. To date, a comprehensive study on the HER performances of MX_2_ monolayers as well as its origin has not been available. In this work, the applications of MX_2_ monolayers in electrolysis of water are systematically investigated based on the calculations of density-functional theory (DFT). We predict that the HER performances of MX_2_ monolayers depend on M, X and H-coverage. We show that VS_2_ is comparable to Pt for electrolysis of water at lower H-coverage and its catalytic activity can be further enhanced by improving conductivity.

## Results and discussion

**Figure 1:**
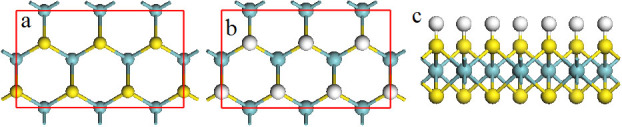
The representative structures of pristine MX_2_ (a) and fully H-covered MX_2_ (b) top and (c) side views.

**Figure 2:**
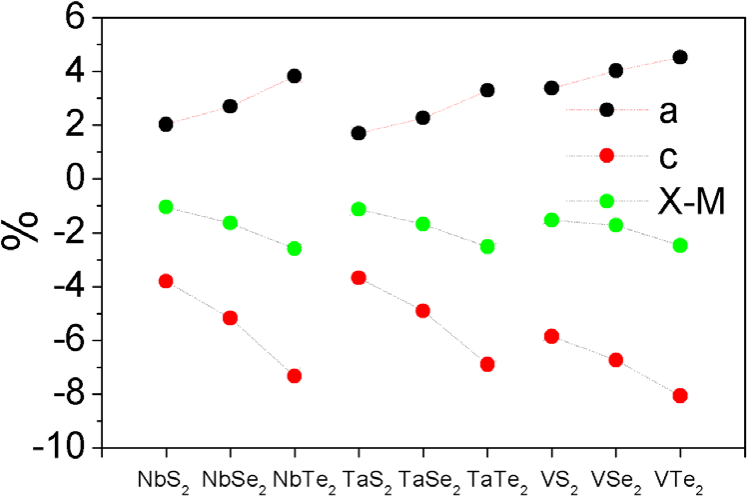
The difference of lattice parameters, including lattice constant (a), monolayer's thickness (c), and X-M bond length (X-M), between pristine and H-covered MX_2_ monolayers.

In our calculations, we focus on metallic/semimetallic 2H transition-metal dichalcogenides, MX_2_ (M = Nb, Ta, and V; X = S, Se, and Te) because 2H phase is more stable and high conductivity is essential to the electrolysis of water. The MX_2_ unit cells with trigonal prismatic (2H) coordination are first optimized to obtain the lattice parameters. The optimized structures of MX_2_ (M = Nb, and Ta; X = S, Se, and Te) (see Supporting Data, Table I) from our calculations are consistent with the reported experimental and theoretical data^46^. We see that the lattice parameters of NbX_2_ are almost equivalent to those of TaX_2_, while are larger than those of VX_2_ (Supporting Data, Table I). To investigate the HER performances of MX_2_ monolayers at various hydrogen coverage on their surfaces, the geometries of MX_2_ monolayers with one surface fully covered by hydrogen atoms (MX_2_-H) are relaxed to find out the effects of hydrogen coverage on their lattice parameters. The hydrogen atoms are adsorbed on the top of X atoms (Figure 1), where is the most stable position^37^,^47^,^48^. The optimized structures of MX_2_-H (Table II in Supporting Data & Figure 2) show that the lattice constants (a) are extended by 2.0 to 3.8%, 1.7 to 3.3%, and 3.4 to 4.5% for NbX_2_, TaX_2_, and VX_2_, respectively, where the extension increases as X changes for S to Se, further to Te (Figure 2). The thicknesses (c) and the X-M bonds of the monolayers are reduced by the H-coverage on their surfaces (Figure 2). The calculated bond lengths are about 1.4, 1.5 and 1.7 Å for S-H, Se-H, and Te-H bonding, respectively (Supporting Data, Table II).

According to the Sabatier principle, the optimal catalytic activity of material for HER can be achieved on a catalytic surface with intermediate binding energies (or free energies of adsorption) for reactive intermediates^15^, which can be quantified by analyzing the reaction free energy of hydrogen adsorption (ΔG_H_)^13^,^15^,^37^,^49^. The optimum value is around ΔG_H_ = 0. To obtain the reaction free energy, we calculate ΔG_H_ for various H coverage on MX_2_ monolayers as following: 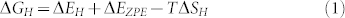
where Δ*E_H_* is the hydrogen chemisorption energy defined as: 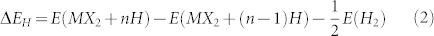
where *n* is the number of H atoms adsorbed on a MX_2_ monolayer. Full coverage refers to one hydrogen atom per X atom adsorbed on one side of the MX_2_ monolayer. The *ΔG_H_* as a function of the hydrogen coverage can be obtained by changing *n*. *E*(*MX_2_* + *nH*), *E*(*MX_2_*) and *E*(*H_2_*) in Eq. (2) are the energies of monolayer with hydrogen atoms (n), pure MX_2_ monolayer (n = 1), and hydrogen molecule, respectively. Δ*S_H_* is the difference in entropy. The entropy of adsorption of 1/2 H_2_ is 
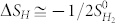
, where 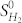 is the entropy of H_2_ in the gas phase at standard conditions. Δ*E_ZPE_* is the difference in zero point energy between the adsorbed and the gas phase, related to the reaction: 1/2*H*_2_(*g*) → H*, where H* denotes a hydrogen atom adsorbed on the surface. Δ*E_ZPE_* − *T*Δ*S_H_* is about 0.24 eV^13^,^37^,^49^, simplifying Eq. (1) to Δ*G_H_* = Δ*E_H_* + 0.24.

**Figure 3:**
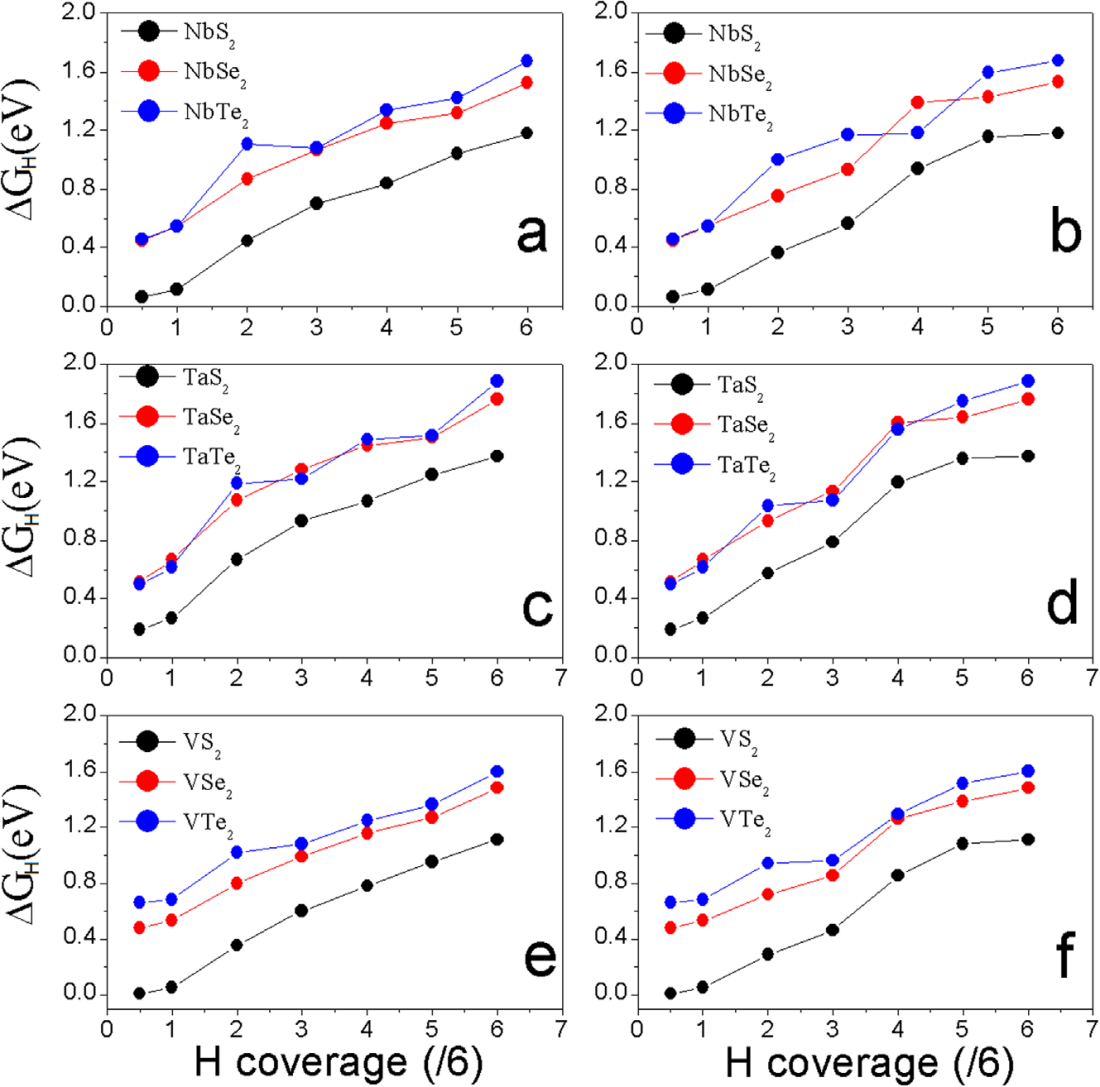
The calculated overpotentials as a function of H-coverage of MX_2_ monolayers in p-supercell by “average” method: (a), (c), and (e); and by “most stable” method: (b), (d) and (f).

**Figure 4:**
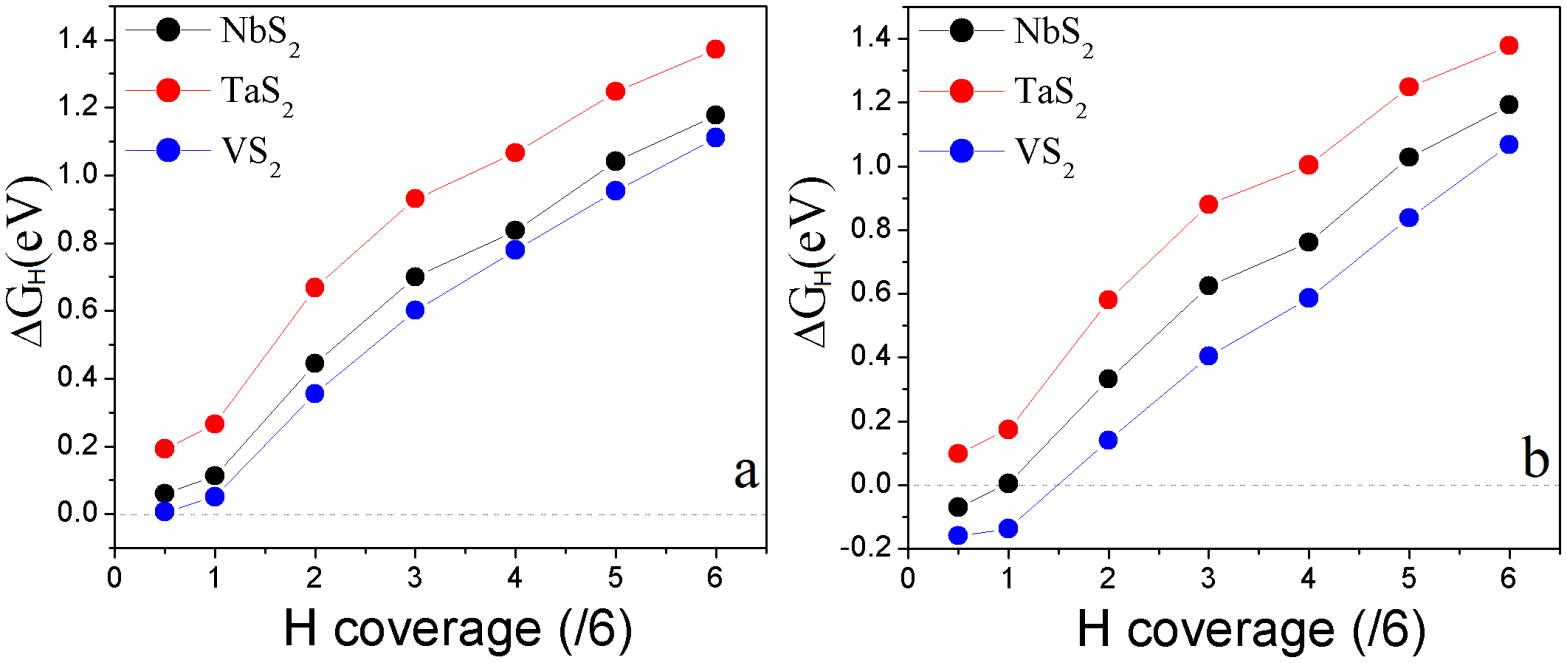
The calculated overpotentials as a function of H-coverage of MS_2_ monolayers by “average” method in p-supercell (a) and in h-supercell (b).

Rectangle supercell (Figure 1) is employed to study the different hydrogen coverage on MX_2_ monolayer, 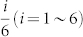. The full coverage (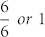) is that all of X atoms on one side of the monolayer adsorb hydrogen atoms (Figures 1b&1c). A supercell with double size in y direction of Figure 1 is used for 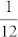 coverage, that is, one H atom is attached to one of the 12 X atoms. 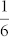 coverage is that one H atom is attached to one of the 6 X atoms (Figure 1). For other coverage (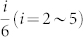), all of the possible H-adsorption configurations on the six X atoms are considered, where the adsorption energy is calculated by two methods – “average” of all configurations and “most stable” configuration. We first consider the supercell (p-supercell) based on monolayer pristine lattice parameters (from Table I in Supporting Data). The calculated adsorption energies show that the overpotentials are positive, and *ΔG_H_* increases with the increase of H coverage on the MX_2_ monolayer (Figure 3), which is in contrast with that of Pt (*ΔG_H_* trends on zero with the increase of H coverage)^13^,^15^,^49^. We see that the calculated *ΔG_H_* follows the same trend regardless of the calculation methods (Figures 3a, 3c, and 3e for “average” method, and Figures 3b, 3d, and 3f for “most stable” method) and the difference on values induced by the method is minor. Importantly, the *ΔG_H_* of MS_2_ is lower than those of MSe_2_ and MTe_2_ by 40% at the same H coverage (Figure 3), indicating that the HER performances of MS_2_ monolayers are better than those of MSe_2_ and MTe_2_ monolayers. The overpotentials at 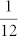 coverage from “average” method (Figures 3a, 3c, & 3e) are 0.061, 0.192, and 0.007 eV for NbS_2_, TaS_2_, and VS_2_, respectively. At 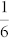 coverage, the *ΔG_H_* for NbS_2_, TaS_2_, and VS_2_ are 0.112, 0.266, and 0.051 eV, respectively. We see that the HER performances of NbS_2_, TaS_2_, and VS_2_ monolayers are better than that of 1T-WS_2_ monolayer because their *ΔG_H_* at relatively high H-coverage is lower than that of 1T-WS_2_ at lower H-coverage (*ΔG_H_* = 0.28 eV at 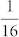 coverage and *ΔG_H_* = 0.36 eV at 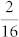 coverage)^37^. The HER performance of VS_2_ at a coverage up to 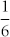 is also better than that of MoS_2_ edges (*ΔG_H_* = 0.08 eV)^28^. For comparison, we compose the curves of *ΔG_H_* of MS_2_ (M = Nb, Ta, and V) monolayers as a function of H-coverage into Figure 4a. We see that the HER performances of MS_2_ monolayers increase as M changes from Ta to Nb, further to V. The overpotential of VS_2_ at 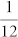 coverage is 10 and 30 times lower than those of NbS_2_ and TaS_2_. The VS_2_-based catalyst for electrolysis of water may be comparable to that of Pt at lower H-coverage (up to 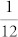 coverage) because of its near-zero overpotential (0.007 eV). The difference at HER performance, however, decreases with the increase of H-coverage on the monolayer. At full coverage (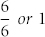), VS_2_ only take advantage of NbS_2_ and TaS_2_ by 6 and 20%, respectively.

**Figure 5:**
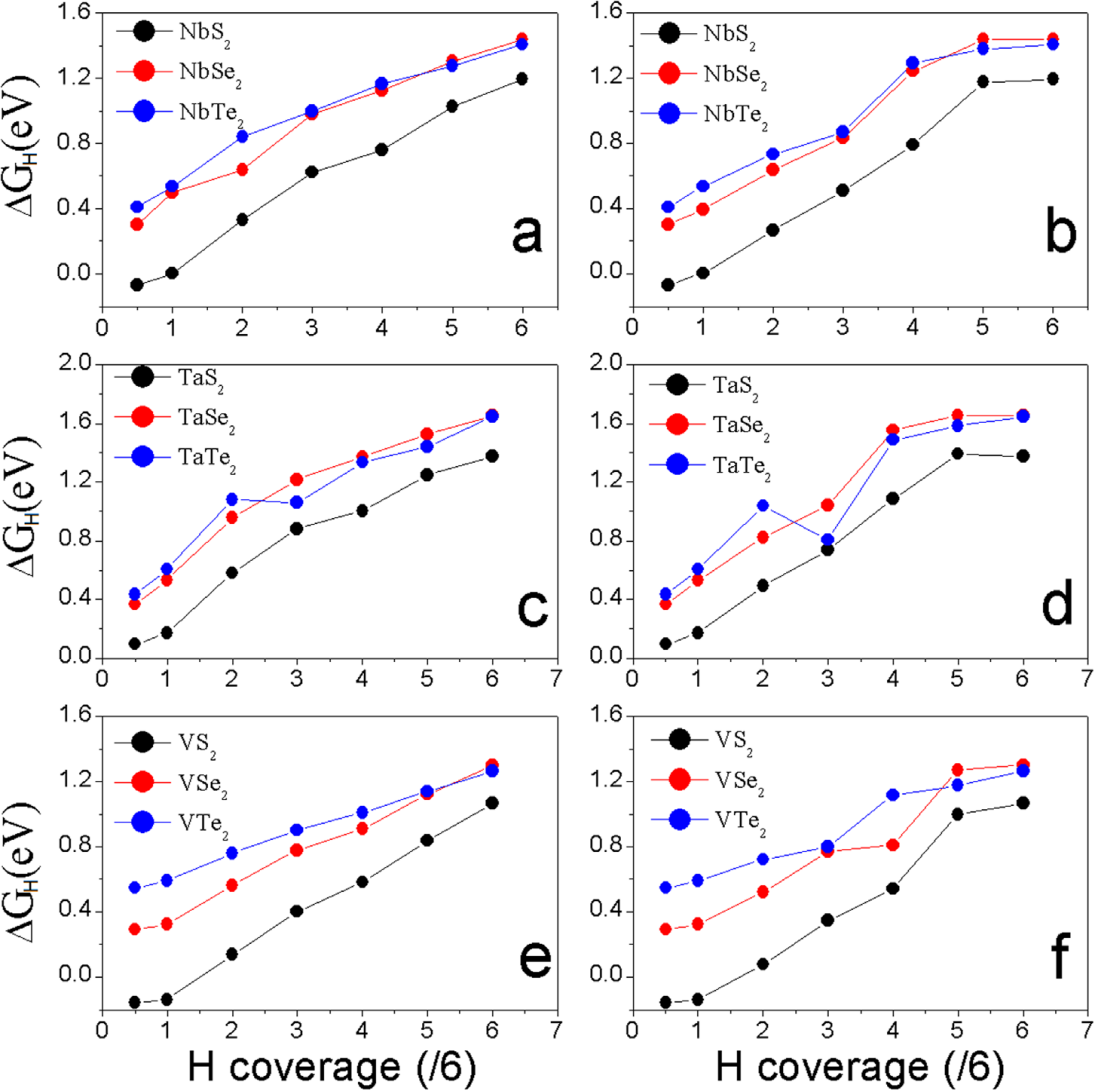
The calculated overpotentials as a function of H-coverage of MX_2_ monolayers in h-supercell by “average” method: (a), (c), and (e); and by “most stable” method: (b), (d) and (f).

**Figure 6:**
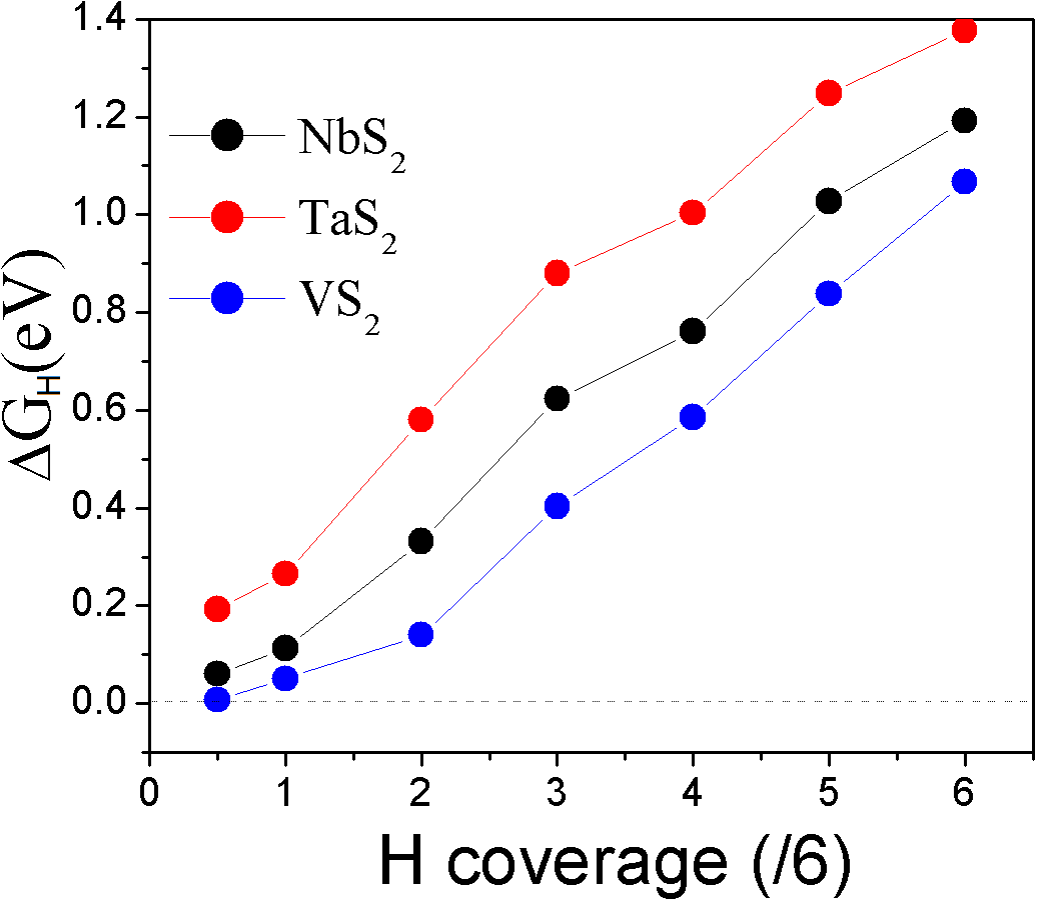
The combined overpotentials as a function of H-coverage of MS_2_ monolayers by “average” method in p-supercell (H-coverage < 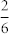) and in h-supercell (H-coverage 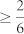).

We also calculate the hydrogen adsorption energy in the supercell (h-supercell) constructed on the lattice parameters of fully H-covered monolayer (from Table II in Supporting Data). We see that the HER trend as a function of H-coverage (Figure 5) is the same as that in p-supercell. The MS_2_ monolayers still show the best HER performance at same H-coverage because their overpotentials are smaller than those of MSe_2_ and MTe_2_ monolayers (Figure 5). For all of MX_2_ monolayers, *ΔG_H_* in h-supercell is reduced because of the extended lattice constants (that is equivalent to the effect of strain in Ref. [37]). The difference of *ΔG_H_* between p-supercell and h-supercell, however, becomes smaller as the H-coverage increases, indicating that the strain shows less effect on HER of the monolayer if the H-coverage is high. At 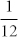 coverage, the overpotentials from “average” method (Figures 5a, 5c, & 5e) are −0.07, 0.098, and −0.159 eV for NbS_2_, TaS_2_, and VS_2_, respectively, and at 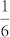 coverage, the corresponding *ΔG_H_* are 0.004, 0.174, and −0.138 eV, respectively, which should imply that NbS_2_ shows the best HER performance at lover H-coverage in h-supercell. With the increase of H-coverage on the MS_2_ monolayers' surfaces, *ΔG_H_* increases (Figure 4b). After 25% H-coverage, VS_2_ gives out the best HER performance because its overpotential is positive and lower than those of NbS_2_ and TaS_2_ (Figure 4b). By carefully comparing the energies of systems calculated from p-supercell and h-supercell, we find that the effect of H-coverage on lattice parameters is negligible when it is less than the 33% (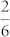
coverage). Therefore, we combine the calculated *ΔG_H_* of MS_2_ from p-supercell for H-coverage up to 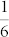 and h-supercell for H-coverage from 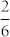 to 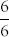 into Figure 6. Clearly, we see that VS_2_ monolayer shows the best HER performance in all of considered systems.

**Figure 7:**
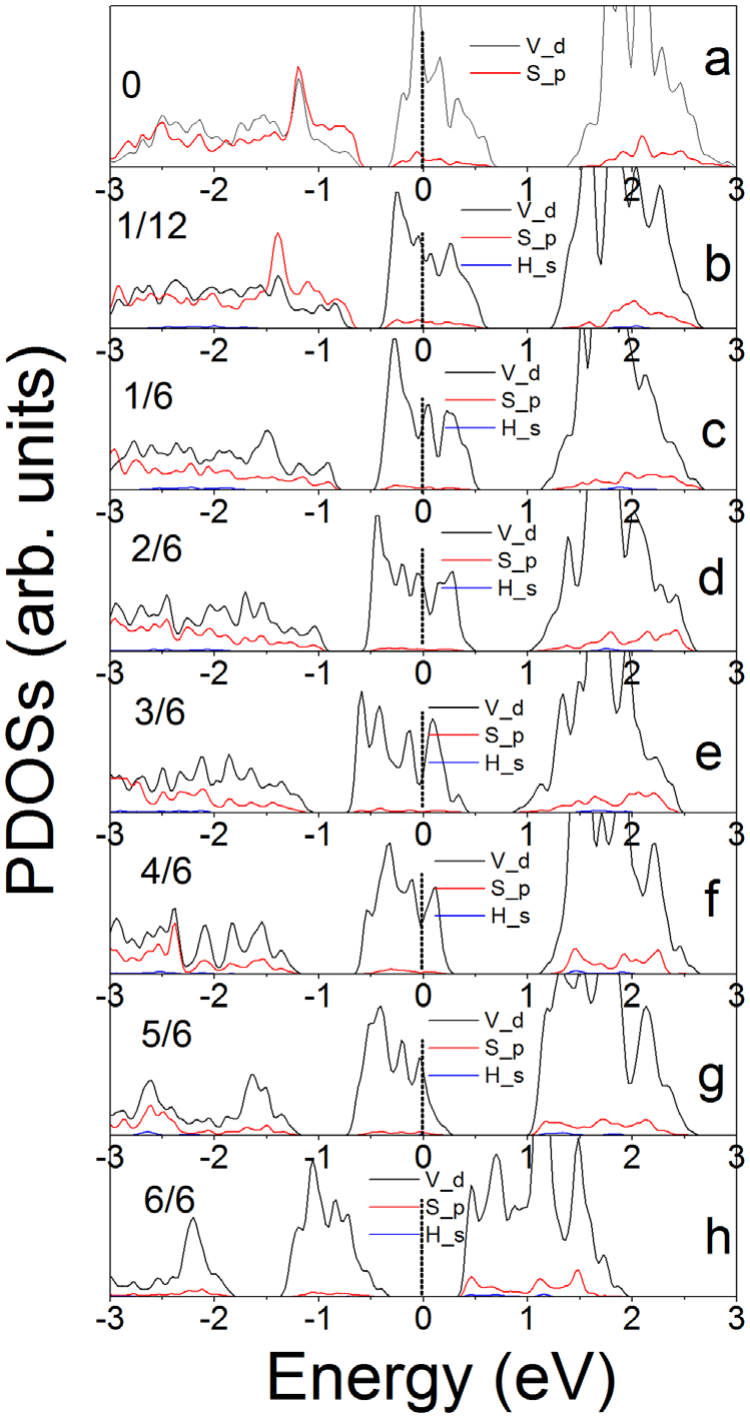
The calculated partial densities of states of pristine (a) and various H-covered (b ~ h) VS_2_ monolayers in p-supercell.

To investigate the possible origin of HER performance and the effects of H-coverage, the partial densities of states (PDOSs) of M, X, and H are calculated. The calculated PDOSs clearly show the change of conductivities of MS_2_ monolayers with the H-coverage (Figure 7 and supporting data S1 ~ S5). We see that the pristine VS_2_ monolayer is metallic, where the Fermi level is within the middle energy band and near to its bottom (Figure 7a). The middle energy states are dominated by V *d* electrons. With introducing H atoms to the monolayer's surface, the energy sates shift down to lower energy region, or the Fermi level shifts up to high energy region within the middle band (Figure 7b). The Fermi level further shifts up with increasing H-coverage (Figure 7c ~ h). At 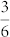 coverage, the Fermi level of the system is close to the middle energy band top. Corresponding to semiconductor's band structure, we may state the middle band as valence band and the band above as conduction band. The up-shift of Fermi level to the middle band (valence band) top leads to the reduction of conductivity. This is further illustrated on VS_2_ monolayer with full H-coverage, where the system is an intrinsic semiconductor or insulator because its Fermi level is within the band gap (Figure 7h). The PDOSs of NbS_2_ and TaS_2_ in p-supercell and h-supercell, and VS_2_ in h-supercell reveal the same origin of the reduction of performance under high H-coverage (Supporting data, S1 ~ S5). The analysis of PDOSs shows that the conductivities of the MX_2_ monolayers are reduced by the H-coverage, especially after half H-coverage on the surfaces, leading to the decreases of their HER performances (Figures 3 ~ 6). This finding may also apply to the MX_2_ nanostructures with metallic edges because their conductivities should be reduced when edges are fully saturated by hydrogen atoms^44^. This is different from Pt-related catalysts, whose conductivities have not been affected by the H-coverage on their surfaces^13^,^15^,^49^. How to improve the conductivities of MS_2_-based catalysts under high H-coverage should be a challenge for their application in electrolysis of water. One of ways is to mix these MS_2_ monolayers with high conducting nanostructures, such as graphene^33^,^50^.

For comparison, the HER performance of MoS_2_ monolayer is calculated. We see that its overpotential (*ΔG_H_*) (Supporting data, S6) is larger than those of MX_2_ (M = Nb, Ta, and V; X = S, Se, and Te) monolayers considered in this work (Figure 6). At lower and full H-coverage (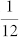,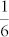, and 1), its overpotential is about 2.0 ~ 2.1 eV. At moderate H-coverage, its overpotential is larger than 1.1 eV. The calculated densities of states show that pure and H-covered MoS_2_ monolayers are intrinsic or n-type semiconductors (Supporting data, S7). These results confirm that the surface of MoS_2_ monolayer is inert to electrolysis of water, and the most active sites are at the edges or defects of MoS_2_ nanostructures^27^,^28^,^29^. However, our study show that the surfaces of MX_2_ (M = Nb, Ta, and V; X = S, Se, and Te) monolayers, especially VS_2_, are very active, which further enhance their ability for electrolysis of water due to higher contacting area with water.

## Conclusions

The DFT-based first-principles calculations are carried out to investigate the hydrogen evolution reduction of MX_2_ monolayers. We find that MS_2_ monolayers are better than MSe_2_ and MTe_2_ monolayers in electrolysis of water, especially VS_2_, which shows the best HER performance because of its lower overpotential. We show that the HER performances of MX_2_ monolayers strongly depend on the H-coverage on their surfaces. With increasing H-coverage, the performance is reduced because of the reduction of conductivity. We also show that the strain may improve the HER performance at relatively low H-coverage. We further predict that their HER applications at high H-coverage can also be achieved by improving their conductivities, such as hybridization with metallic nanostructures. It is expected that the MS_2_ monolayers, especially VS_2_, may find applications to electrolysis of water for hydrogen production.

## Methods

The first-principles calculations are carried out to investigate the hydrogen evolution reduction of transition-metal dichalcogenide monolayers. Our calculations are based on the density functional theory (DFT)^51^ and the Perdew-Burke-Eznerhof generalized gradient approximation (PBE-GGA)^52^. The projector augmented wave (PAW) scheme^53^,^54^ as incorporated in the Vienna ab initio simulation package (VASP)^55^ is used in the study. The Monkhorst and Pack scheme of k point sampling is used for integration over the first Brillouin zone^56^. A 15 × 15 × 1 grid for k-point sampling for geometry optimization of unit cells, and an energy cut-off of 450 eV are consistently used in our calculations. A sufficiently large supercell is used so that the monolayers in neighbouring cells in the vertical direction are separated by a vacuum region of at least 20 Å. Good convergence is obtained with these parameters and the total energy was converged to 2.0 × 10^−5^ eV/atom. The error bar (or uncertainty) of the DFT calculation is less than 5 meV. The thermodynamic processes via Tafel pathway are calculated^17^. The effect of solvent on the HER performance of VS_2_ monolayer is investigated by including H_2_O molecules in the systems with various H-coverage^18^,^21^.

The calculated thermodynamc processes of HER on VS_2_ monolayer via Tafel channels and the effect of solvent on overpotentials are included in Supporting Data.

## Author Contributions

H.P. conceived the idea, performed the calculations, and wrote the paper.

## Supplementary Material

Supplementary Information Supporting data
