# Supplementary material for: Metal Dichalcogenides Monolayers: Novel Catalysts for Electrochemical Hydrogen Production
Source: Sci Rep. 2014 Jun 26;4:5348. doi: 10.1038/srep05348 (PMC4071773; doi:10.1038/srep05348)
Supplement: Supplementary Information — Supporting data [file srep05348-s1.pdf]

Metal dichalcogenides monolayers: Novel catalysts for electrochemical hydrogen evolution

Supporting Data

Hui Pan

Institute of Applied Physics and Materials Engineering, Faculty of Science and Technology, University of Macau, Macau SAR, China

	a (Å)	c (Å)	X-M (Å)	∠X-M-X (^{o})	∠M-X-M (^{o})
NbS_2	3.343	3.124	2.483	84.61/77.99	84.61
$NbSe_2$	3.473	3.360	2.616	83.18/79.93	83.18
$NbTe_2$	3.683	3.692	2.816	81.69/81.92	81.69
TaS_2	3.342	3.128	2.483	84.57/78.06	84.57
$TaSe_2$	3.477	3.359	2.617	83.17/79.86	83.17
$TaTe_2$	3.703	3.665	2.816	82.26/81.18	82.26
VS_2	3.167	2.971	2.356	84.45/78.19	84.45
VSe_2	3.326	3.190	2.496	83.49/79.16	83.49
VTe_2	3.572	3.507	2.707	82.57/80.74	80.47

Table I, Lattice parameters of MX_2 (M = Nb, Ta, and V; X = S, Se, and Te)

	a (Å)	c (Å)	X-M (Å)	∠X-M-X (^{o})	∠M-X-M (^{o})	X-H (Å)
NbS_2-H	3.411	3.005	2.457	87.93/74.67	87.93	1.364
$NbSe_2$-H	3.567	3.186	2.573	87.77/75.42	87.77	1.506
$NbTe_2$-H	3.824	3.421	2.743	85.38/75.49	85.38	1.710
TaS_2-H	3.399	3.013	2.455	87.63/75.02	87.63	1.366
$TaSe_2$-H	3.556	3.194	2.573	87.43/75.74	87.43	1.509
$TaTe_2$-H	3.825	3.412	2.745	88.33/75.34	88.33	1.714
VS_2-H	3.274	2.797	2.320	89.75/72.96	89.75	1.370
VSe_2-H	3.460	2.975	2.453	89.68/73.32	89.68	1.514
VTe_2-H	3.734	3.224	2.640	90.04/73.51	90.04	1.719

Table II, Lattice parameters of MX_2 with one surface fully covered by hydrogen atoms (M = Nb, Ta, and V; X = S, Se, and Te)

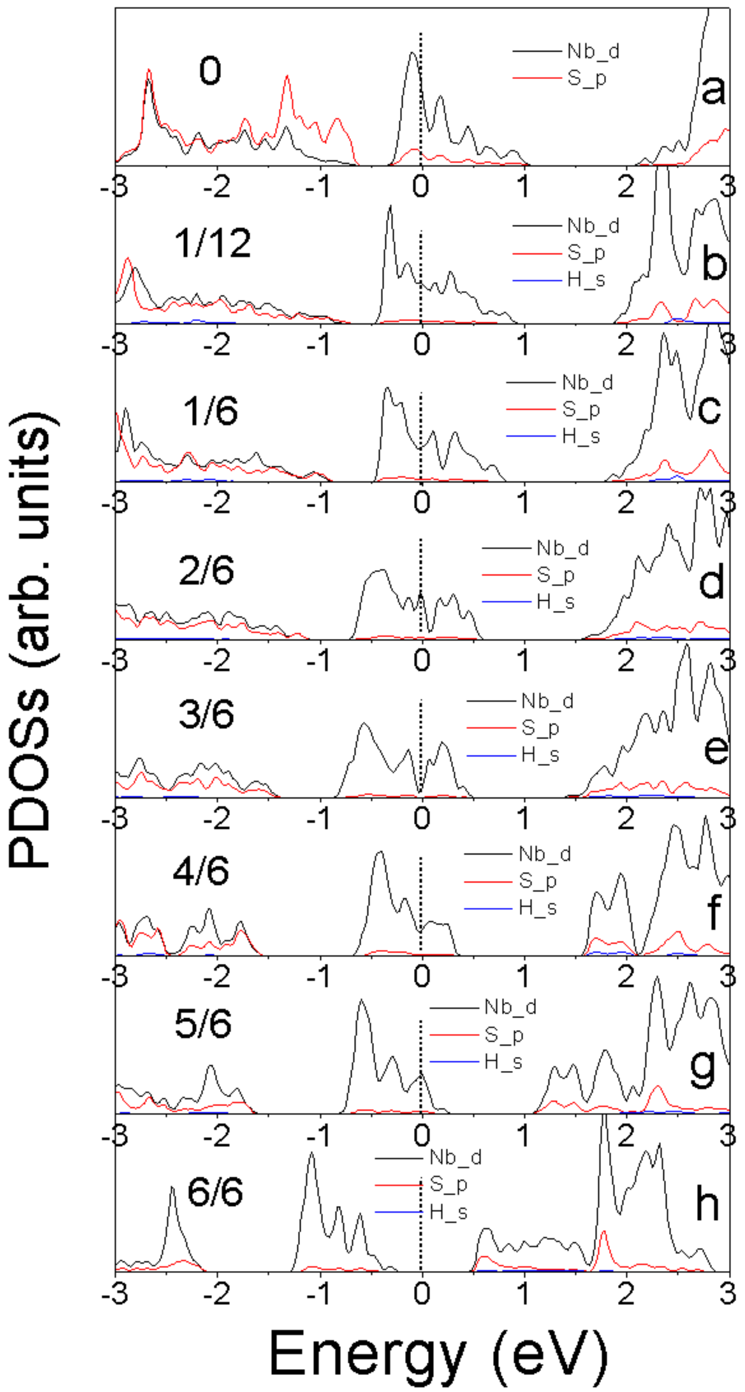

S1, Calculated partial densities of states of pristine (a) and various H-covered (b~h) NbS_2 monolayers in p-supercell.

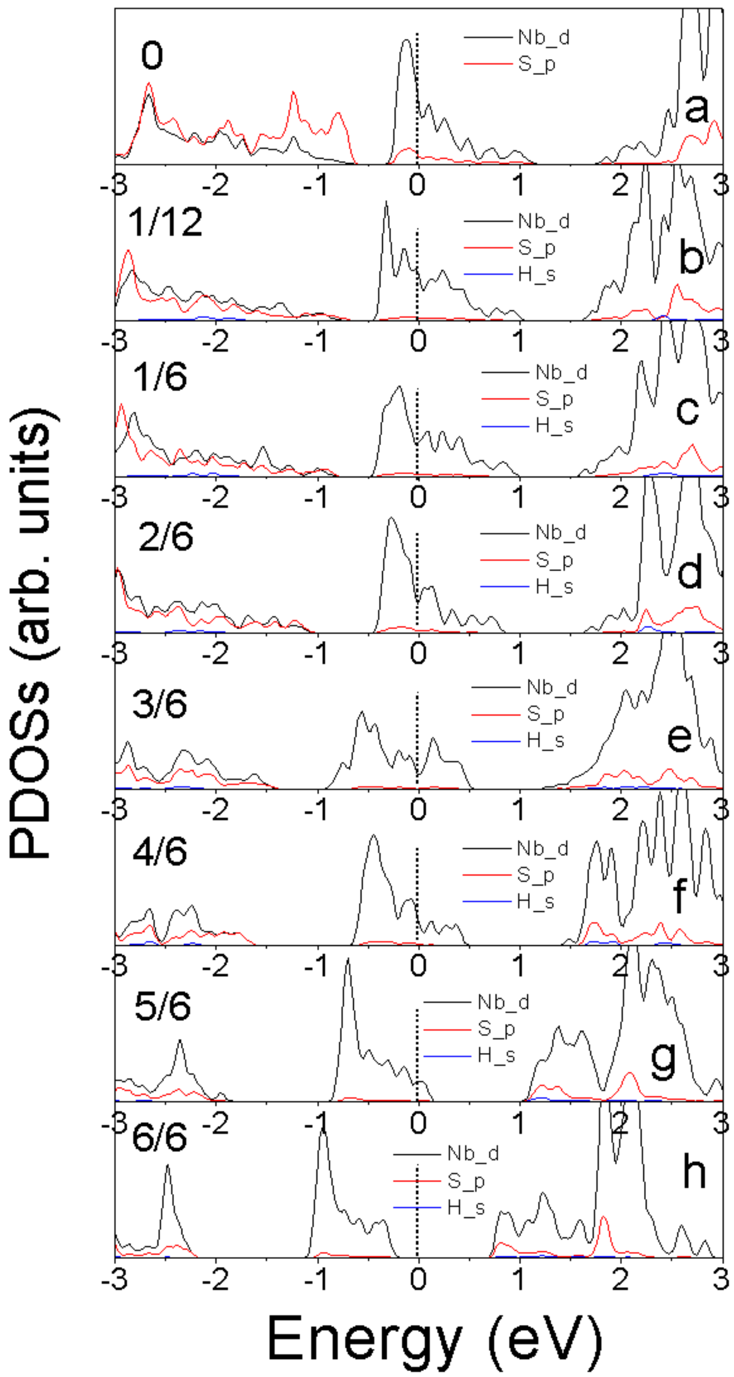

S2, Calculated partial densities of states of pristine (a) and various H-covered (b~h) NbS_2 monolayers in h-supercell.

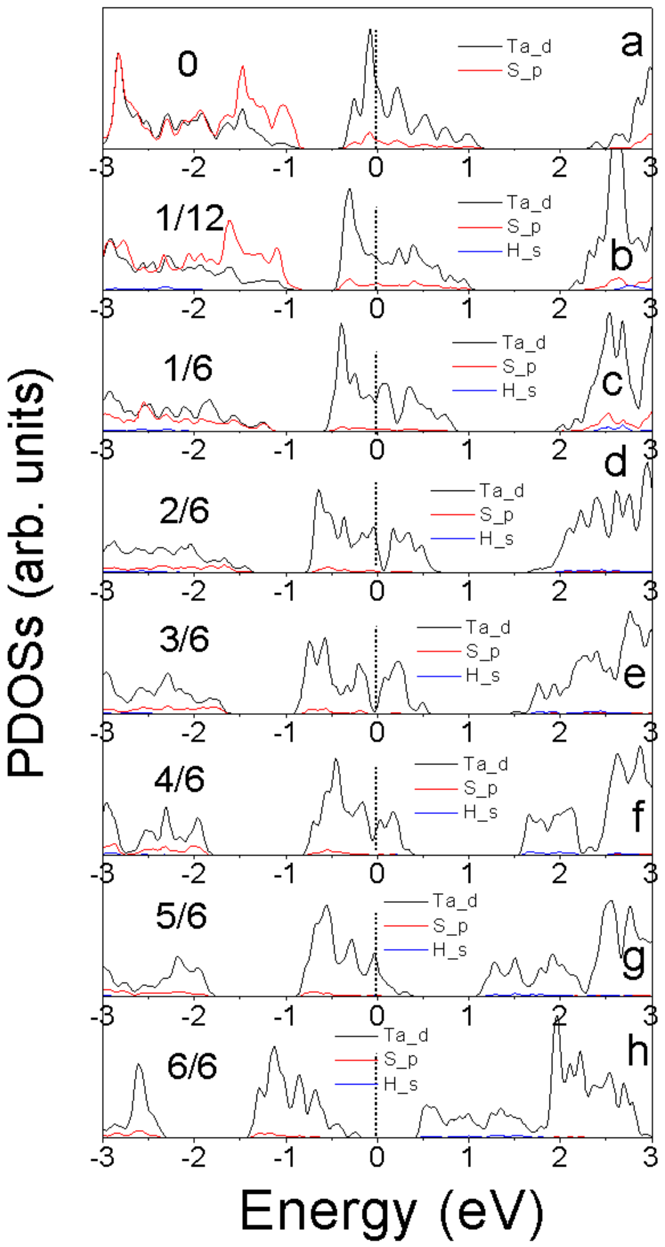

S3, Calculated partial densities of states of pristine (a) and various H-covered (b~h) TaS_2 monolayers in p-supercell.

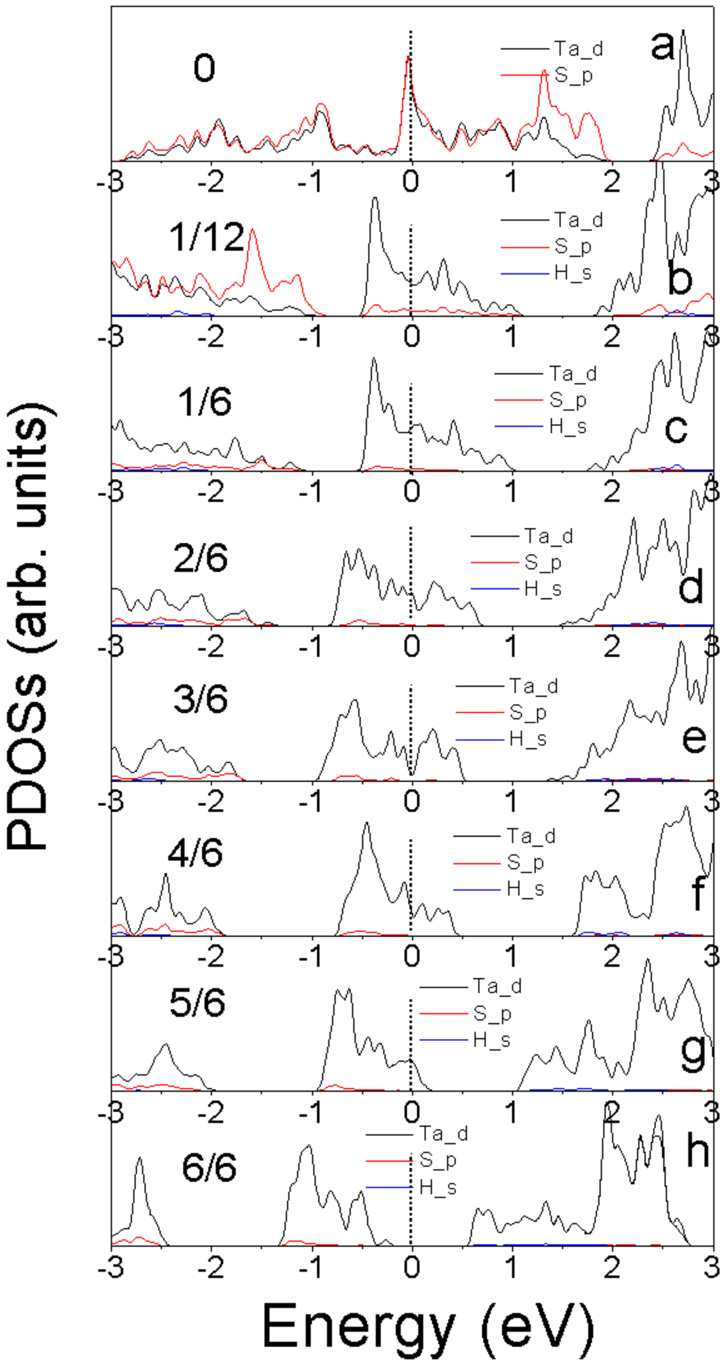

S4, Calculated partial densities of states of pristine (a) and various H-covered (b~h) TaS_2 monolayers in h-supercell.

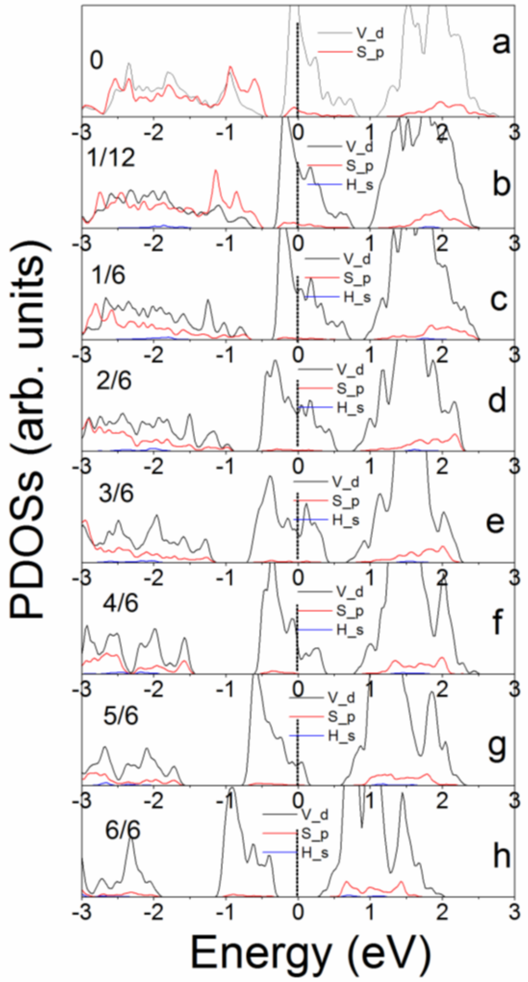

S5, Calculated partial densities of states of pristine (a) and various H-covered (b~h) VS_2 monolayers in h-supercell.

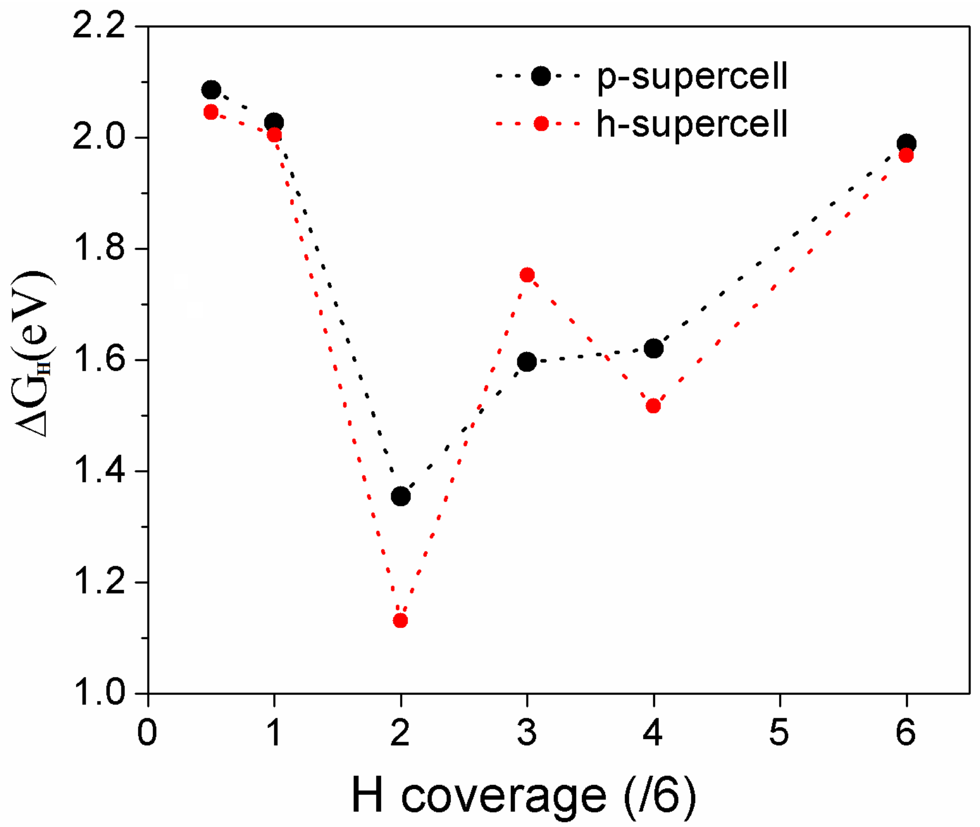

S6, Calculated overpotentials as a function of H-coverage of MoS_2 monolayers by "most stable" method in p-supercell and h-supercell. The overpotential at a H coverage of 5/6 is unavailable because all of the coverage configurations are not stable, and that at 6/6 is estimated by $\Delta E_H = (E(MX_2 + nH) - \frac{n}{2}E(H_2))/n$, indicating that the surface of MoS_2 monolayer is not suitable for hydrogen evolution reaction at high H-coverage.

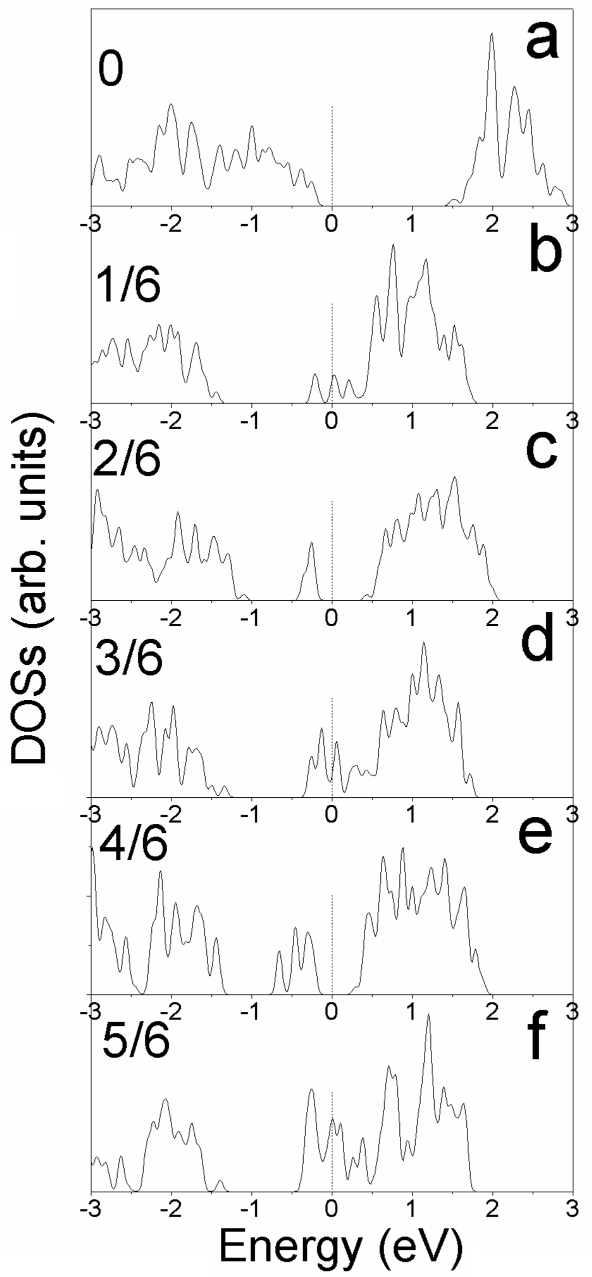

S7, Calculated densities of states of pristine (a) and various H-covered (b~f) MoS_2 monolayers in h-supercell. Fermi levels are at 0 eV. The pure MoS_2 monolayer is an intrinsic semiconductor with a gap of 1.8 eV and H-covered MoS_2 systems are intrinsic or n-type semiconductors, revealing the origins of their high overpotentials.

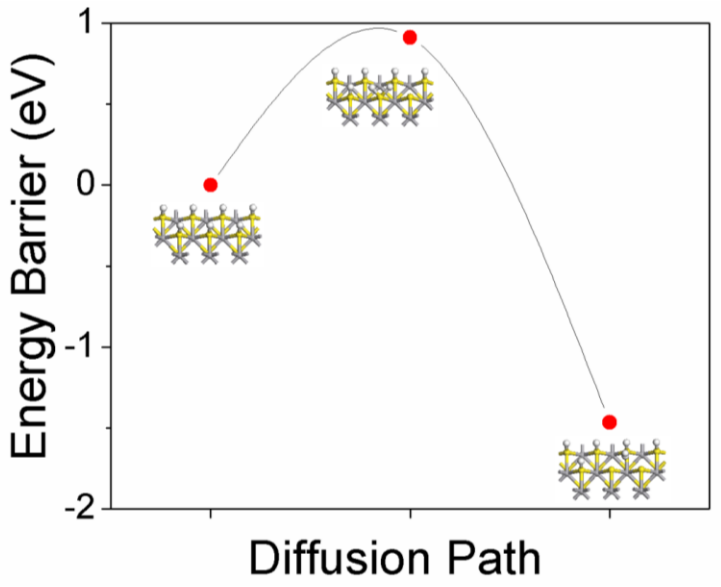

S8, Calculated energy profile via Tafel pathway for hydrogen evolution reaction on VS_2 monolayer at full H-coverage. The insets show initial, transitional, and final states from left to right.

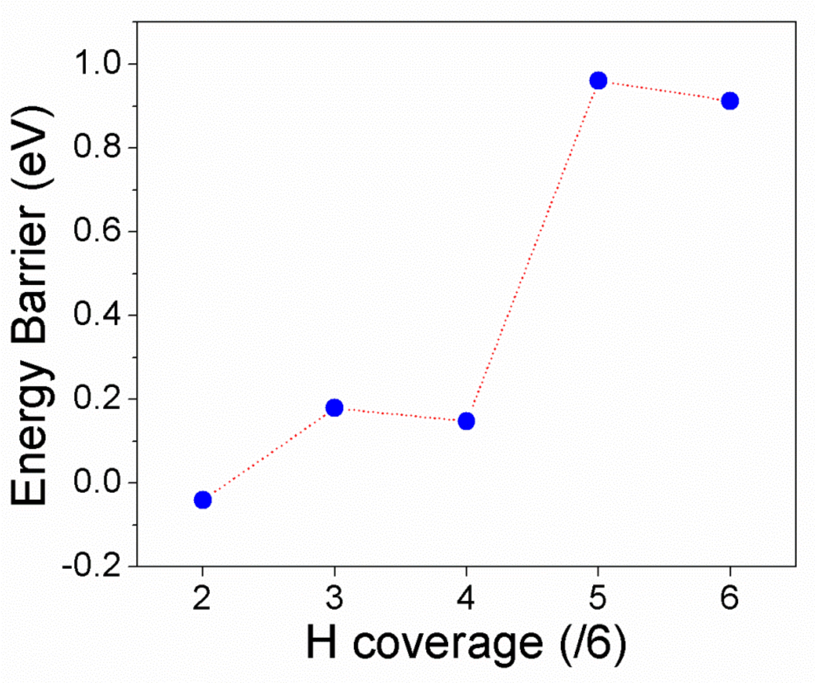

S9, Calculated energy barriers of for hydrogen evolution reaction on VS_2 monolayer via Tafel pathway at various H-coverage ($\frac{2}{6}$ to $\frac{6}{6}$). The energy barrier increases with increasing H-coverage, consistent with the calculated overpotential as a function of H-coverage.

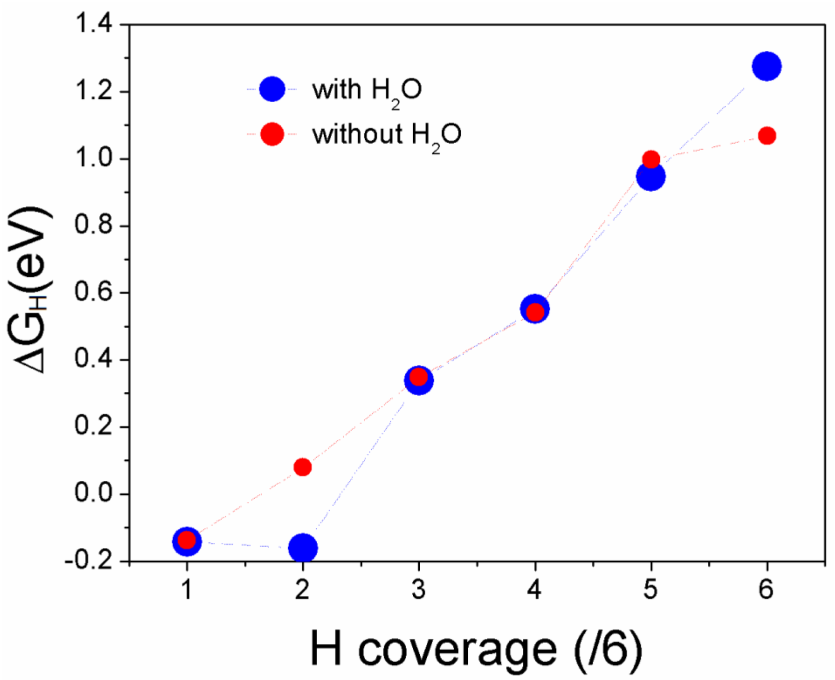

S10, Calculated effect of solvent on HER performance of VS_2 monolayer at various H-coverage ($\frac{1}{6}$ to $\frac{6}{6}$). We see that solvent has negligible effect on HER performance of VS_2 monolayer, except that at full H-coverage, where the HER ability is reduced because of increased overpotential.
